# Tunable Microwave Filters Using HfO_2_-Based Ferroelectrics

**DOI:** 10.3390/nano10102057

**Published:** 2020-10-18

**Authors:** Martino Aldrigo, Mircea Dragoman, Sergiu Iordanescu, Florin Nastase, Silviu Vulpe

**Affiliations:** National Institute for Research and Development in Microtechnologies (IMT Bucharest), Erou Iancu Nicolae Street 126A, 077190 Voluntari, Ilfov, Romania; martino.aldrigo@imt.ro (M.A.); sergiu.iordanescu@imt.ro (S.I.); florin.nastase@imt.ro (F.N.); silviu.vulpe@imt.ro (S.V.)

**Keywords:** microwaves, tunable filter, hafnium oxide, ferroelectric material

## Abstract

In this paper, we present microwave filters that are based on 6-nm-thick ferroelectric thin films of hafnium oxide doped with zirconium (HfZrO), which are tunable continuously in targeted bands of interest within the frequency range 0.1–16 GHz, when the applied direct current (DC) voltage is swept between 0 V and 4 V. Here, we exploit the orthorhombic polar phase in HfO_2_ through a careful doping using zirconium in an Atomic Layer Deposition (ALD) process, in order to guarantee phase stabilization at room temperature. Polarization versus voltage characterization has been carried out, showing a remanent polarization (*P_r_*) of ~0.8 μC/cm^2^ and the coercive voltage at ~2.6 V. The average roughness has been found to be 0.2 nm for HfZrO films with a thickness of 6 nm. The uniform topography, without holes, and the low surface roughness demonstrate that the composition and the structure of the film are relatively constant in volume. Three filter configurations (low-pass, high-pass, and band-pass) have been designed, modelled, fabricated, and fully characterized in microwaves, showing a frequency shift of the minimum of the reflection coefficient between 90 MHz and 4.4 GHz, with a minimum insertion loss of approximately 6.9 dB in high-pass configuration.

## 1. Introduction

Hafnium oxides (HfO_2_) have been gathering momentum in the very last years, thanks to their versatility in being exploited for low-power electronics. One of the most appealing characteristics of HfO_2_ is represented by its electrical permittivity, which can be increased while using structural phase transformations [1]. HfO_2_ in its most stable phase, i.e., the monoclinic phase, has the lowest permittivity (*ε_r_* ≈ 18). HfO_2_ exhibits a much higher permittivity in other phases, such as cubic (*ε_r_* ≈ 27) or tetragonal (*ε_r_* ≈ 70), but these phases are stable at temperatures higher than 1700 °C. The increasing of the permittivity of HfO_2_ is of outmost importance in decreasing the effective oxide thickness (EOT) and, thus, the leakage current, in CMOS transistors when billions of them are integrated in a single silicon (Si) chip. The solution to decrease the temperature in order to obtain a phase transition is the doping of very thin films of HfO_2_. The cubic phase was attained by doping the HfO_2_ with yttrium (Y) [1], while the tetragonal phase was obtained by doping with Si [2]. However, the doping with ZrO_2_ of HfO_2_ that was grown by atomic layer deposition (ALD) [3,4] has demonstrated the possibility of achieving the tetragonal phase of HfO_2_ at room temperature. The realization of a high permittivity depends on the concentration of dopants, the annealing temperature, and the growth method; this way, the value of the permittivity of the doped HfO_2_ could be increased by two–three times from *ε_r_* ≈ 18 in the monoclinic phase.

The experience gained during years of studies in the enhancement of HfO_2_ permittivity is the basis of the discovery of the orthorhombic phase that confers ferroelectric properties to HfO_2_. The same dopants were used as above, but in different growth conditions, since the orthorhombic phase requires very strict control of dopant concentration, temperature, and mechanical strain [5,6]. Cubic and tetragonal, as well as orthorhombic polymorphs of HfO_2_, can be stabilized by using different dopants, annealing procedures and layer thicknesses. This was demonstrated by ab-initio studies [7] and many experiments reported in the review paper [8].

Nowadays, the most used HfO_2_-based ferroelectric is the one doped with zirconium (Zr), termed as HfZrO. Can we further enhance the electrical permittivity of HfO_2_-based ferroelectrics? The answer is positive, since, in any ferroelectric, there is a strong dependency of electrical permittivity on the applied voltage, this property being used extensively in the case of high-frequency devices for tuning their main characteristics [9]. We have recently shown that, in a very large band (1–8 GHz), there is an increase of 27% of the permittivity of HfZrO when a DC bias voltage is swept in the range 0–5 V [10]. Based on these results, the aim of this paper is to show that we can tune the reflection (|S_11_| or |S_22_|) and transmission (|S_21_| or |S_12_|) coefficients of a low-pass filter (LPF), a high-pass filter (HPF), and a band-pass filter (BPF) when the applied DC voltage is tuned in the range 0–4 V. These circuits are of outmost importance in many areas of wireless communications where filters are ubiquitous components [11]. In detail, there are some key characteristics of HfZrO thin films that make them a target material for (potential) high-performance tunable filters. First, the proposed ferroelectric requires low values of DC bias voltage in order to modulate its dielectric constant. The nanoscale thickness (in the order of some nm) ensures a very large critical (coercive) electric field *E_c_*. A direct consequence of the latter property is the following: large values of *E_c_* entail that small values of an external DC bias voltage (i.e., in the range ±5 V) are enough to tune ferroelectric’s permittivity. This represents a noteworthy advancement with respect to ‘classic’ ferroelectrics, like perovskites (PZTs), as tunable microwave circuits that are based on them need bias values in the order of tens of volts. Second, a full CMOS compatibility is guaranteed, which allows large-scale fabrication. Third, high values of the dielectric constant represent an advantage in designing high-frequency devices with compact dimensions, thanks to a reduction of the guided wavelength. Besides this, the exploitation of ALD techniques gives a further improvement in the overall fabrication process, since it provides precise control of ferroelectric’s thickness, a high homogeneity in terms of surface roughness, and the possibility of using large-area substrates, an undoubtable benefit when testing components for extensive measurements.

## 2. Materials and Methods

The key of the proposed tunable microwave filters is represented by the voltage- and frequency-dependent dielectric constant of the HfZrO thin film. However, the extraction of the permittivity of HfZrO in microwaves, as a function of the applied DC voltage bias, is a non-trivial problem. ‘Traditional’ methods that are used to analyse multilayer interdigital capacitors (IDCs) or coplanar waveguide (CPW) lines (like single layer reduction or microstrip dual resonator) inevitably fail, since the nanometric scale thickness of the HfZrO ferroelectric thin film makes it impossible to apply analytical methods. Hence, starting from the results that were published in [10,12], via a fitting procedure exploiting electromagnetic (EM) simulations and experiments we can derive the following equation, which expresses the dependence of the relative permittivity *ε*(*V*) of the 6-nm-thick HfZrO on the applied voltage, at an assigned frequency:

*ε*(*V*) = [*αV* + *ε_eff_*(0)/(*ε_eff,bulk_*)^1⁄2^]^2^ + *ε_eff_*(0), (1)

where 0 < *α* < 1 V^−1^ (which depends on the given frequency) is a characteristic constant of HfZrO, whereas *ε_eff_*(0) is the effective permittivity at 0 V of the HfZrO/bulk substrate, and *ε_eff,bulk_* is the effective permittivity of the sole bulk substrate. In terms of electric fields:

*ε*(*V*) = [*α*(*E_a_* − *E_c_*)*t* + *ε_eff_*(0)/(*ε_eff,bulk_*)^1⁄2^]^2^ + *ε_eff_*(0), (2)

where *t* is the thickness of the HfZrO thin film, *E_a_* is the external applied DC electric field, and *E_c_* is the coercive field (i.e., 1–2 MV/cm for HfZrO). Equation (2) is derived in a straightforward way from Equation (1), while taking into account that the DC voltage that is applied to the HfZrO thin film is physically determined by both the external contribution of *E_a_* (which can be estimated also by static EM simulations [10,12]) and the intrinsic contribution of *E_c_*, in order to describe the hysteretic behaviour of the permittivity. These simple relations fit well with the experimental results, if one knows *ε_eff_*(0) and *ε_eff,bulk_* (at the desired frequency) that can be easily obtained by either theoretical/simulation results or measurements of scattering parameters. To our knowledge, this approach is the only one feasible for characterizing the high-frequency permittivity of ferroelectric thin films, as other methods take into account either thicker ferroelectrics in vertical capacitor-like configurations or very low frequencies [13,14].

**Figure 1:**
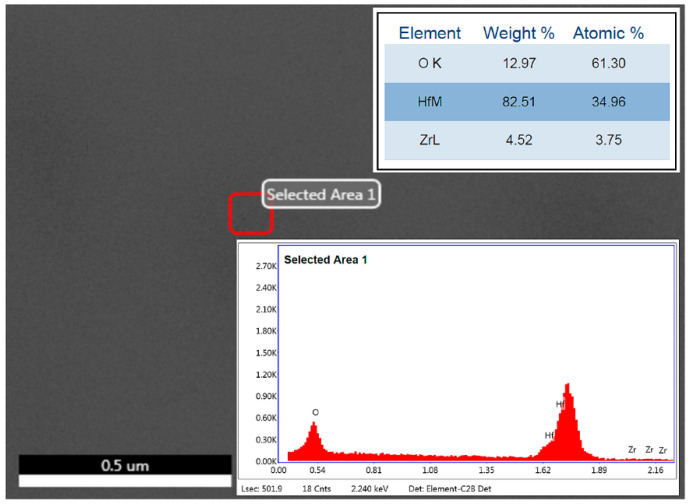
Energy-dispersive X-ray (EDX) analysis spectra of the 6-nm-thick HfZrO film.

The substrate used for the three filters is a 6-nm-thick Zr-doped HfO_2_ thin film grown by ALD technique on a 4-inch wafer of 525-μm-thick high-resistivity (HR) Si. An Oxford Instruments OpAL ALD reactor was used for the growing of ferroelectric thin films while using Tetrakis(dimethylamino)hafnium (TDMAH, 99.99+%-Hf, <0.2%-Zr, Strem Chemicals. Inc., Newburyport, MA, USA) and Tetrakis(dimethylamino)zirconium (TDMAZ, 99.99+%-Zr, Strem Chemicals. Inc., Newburyport, MA, USA) as metal organic precursors. Ultra-pure water was used as the oxidant, and ultra-high purity nitrogen (Nitrogen 6.0, 99.9999 vol.%) was used as the purge and carrier gas. Prior to the ALD process, the silicon wafer was subjected to surface cleaning treatment by exposure to UV-ozone for 15 min. at 75 °C, while using an UV-Ozone cleaner (Novascan Technologies Inc., Ames, IA, USA). The ferroelectric HfO_2_ films were grown by sequentially mixing the two precursors on the silicon wafer surface, in a ratio of 2:1, followed by their oxidation to produce a homogeneous binary oxide. The binary oxide films grown were indexed: HfZrO. The deposition was done with 56 ALD cycles at 200 °C. The film thickness, as measured by X-ray reflectivity (XRR), was found to be around 6 nm. The Ti (10 nm)/Au (360 nm) top metallic circuits (LPF/HPF/BPF) were deposited while using an Electron Beam Evaporation System (Temescal FC2000, Ferrotec, Bedford, NH, USA), and they were photolithographically configured using a standard lift-off process. Due to the low thickness of the HfZrO film, the Energy Dispersive X-ray spectroscopy (EDX) technique can be considered for identifying and quantifying elemental compositions. Figure 1 shows the EDX analysis spectrum of the 6-nm-thick HfZrO film. From the EDX measurements, the film composition was estimated to be HfZrO as Hf_0.73_Zr_0.27_O_1.85_.

**Figure 2:**
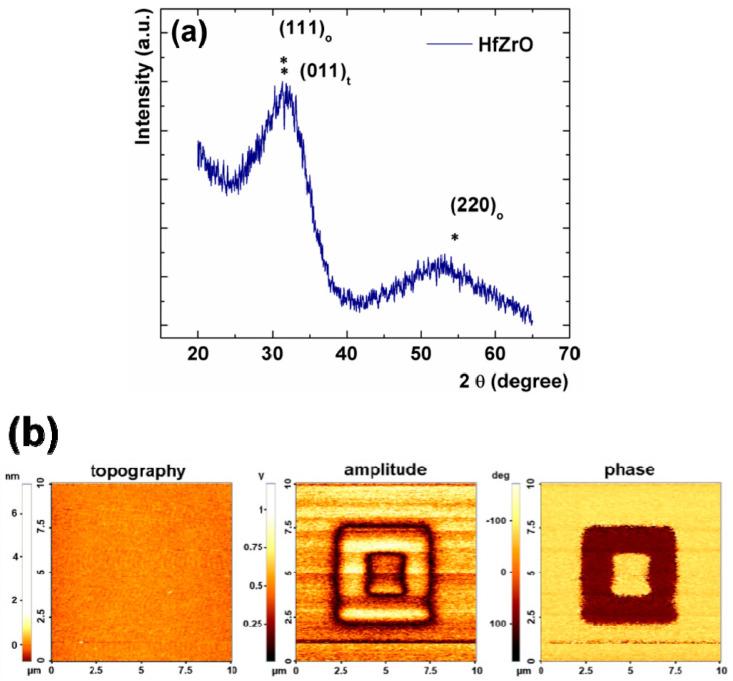
(**a**) Grazing incidence X-ray diffraction (GIXRD) spectra and (**b**) piezo-response force microscopy (PFM) topography (left), amplitude (centre), and phase (right) of the 6-nm-thick HfZrO film.

Grazing incidence X-ray diffraction (GIXRD) and piezo-response force microscopy (PFM) measurements were used in order to validate the ferroelectricity of the HfZrO thin film. GIXRD measurements were performed while using a high-resolution X-ray diffractometer that employs Cu Kα1 radiation (*λ* = 1.54 Å). The incidence angle was set to 0.35 degrees. Figure 2a shows the GIXRD spectra of the HfZrO thin film in the wide 2θ range of 20–65°, and indicates the coexistence of several phases in Zr-doped HfO_2_ thin films. Due to the polycrystalline nature of HfZrO layers deposited by the ALD technique, the mixed composition of orientations and phases, as well as the very small films thickness up to ~10 nm, the structural analysis of HfZrO thin films is not an easy task. In addition, even the GIXRD measurements cannot clearly distinguish between the orthorhombic and tetragonal structures of HfO_2_ due to the structural similarities of the orthorhombic and tetragonal phases (similarities in their lattice structures) [5]. The most intense diffraction peak is assigned as the mixture of (011) of the t-phase and (111) of the o-phase, formed by the presence of overlapping reflections that are associated with the tetragonal (P42/nmc) and the orthorhombic polar phase (Pca2_1_) [6]. The formation of the orthorhombic polar phase in Zr-doped HfO_2_ thin films is confirmed by the presence of the two diffraction peaks near 2θ = 30.5° and 55° assigned to (111)_o_ and (220)_o_ reflections. The GIXRD spectrum also indicates an inhibition of m-phase HfO_2_ that was induced by Zr doping, by presence of the significantly lower monoclinic (111)_m_ reflections, expected around 28° at 2θ angle. The inhibition of the monoclinic reflections and the presence of the orthorhombic polar and tetragonal phases indicate that the film has a polycrystalline structure in which the ferroelectric phase is dominant [15]. The absence of peaks due to the Si substrate, in GXRD measurement, can be obtained by changing the orientation of the silicon wafer in the X-ray field, so the signal on the Si substrate is suppressed. PFM measurements were also carried out on the HfZrO thin films to further demonstrate ferroelectricity. For the manipulation of the ferroelectric domains, the atomic force microscopy (AFM) tip was held at 0 V and a DC bias between −10 V and 10 V was applied to the substrate during scanning. The local mechanical response was determined by applying an alternating current (AC) bias to the AFM tip, while the substrate was held at 0 V. Figure 2b displays the topography image together with the corresponding images of the amplitude and phase signals, which show the intensity of the local mechanical response, and the phase shift between the response and the excitation, respectively. The PFM measurements demonstrate that the HfZrO film exhibits an easy switching of polarization along the poling direction, with clear boundaries between upward and downward domains being observed in phase and amplitude images. The phase shift differs by approximately 180° between the successively written areas, showing that the fabricated HfZrO thin film is indeed ferroelectric.

In addition to PFM investigations, average roughness measurements were performed on 2 μm × 2 μm areas. The average roughness was found to be 0.2 nm for HfZrO films with a thickness of 6 nm. The uniform topography, without holes, and the low surface roughness, give information about the composition and structure of the film, which are relatively constant in volume [16].

Perhaps the most recognizable electrical characterization of a ferroelectric is the hysteresis loop. In order to test the ferroelectric behaviour of the HfZrO thin film, we obtained the polarization versus voltage (P–V) loop. During the initial electric field cycling, the remanent polarization (*P_r_*) was found at ~0.8 μC/cm^2^ and the coercive voltage at ~2.6 V. A well-known phenomenon in ferroelectrics is the wake-up effect, which does not appear in the case when HfZrO is grown directly on Si (as in our case) even if the HfZrO has a thickness of 1 nm [17].

We stress here that the ferroelectric phase is assigned to the orthorhombic polar phase (o-phase) corresponding to the Pca2_1_ space group [18], but other phases are generally present inside the hafnium oxide after the growth process, due to stabilization problems. At room temperature, the stable form of the HfO_2_-based compounds is a monoclinic phase (P21/c, m-phase), whereas, at high-temperatures and pressures, the tetragonal (P42/nmc, t-phase) and the cubic (Fm3m, c-phase) phases are dominant [19]. Nevertheless, for hafnium oxide films up to 10–15 nm in thickness, the transition temperatures of the monoclinic to the tetragonal phase are significantly lowered [20]. In addition, the tetragonal and cubic phases can be stabilized at room temperature via doping [21]. Since 2011, when ferroelectricity in Si-doped HfO_2_ thin films was first reported [5], it has been shown that many other dopants, such as Y, Al, Zr, Gd, Yb, Sr, and La, can induce ferroelectricity in the HfO_2_ thin films [22]. Through a suitable combination of doping, stress and/or electric field the tetragonal P42/nmc phase can make a transition into the polar orthorhombic Pca2_1_ phase [23]. Moreover, a remarkable feature is that, as the thickness increases (>10 nm), the ferroelectricity in HfO_2_-based compounds disappears. Hf-based ferroelectrics only have nanoscale ferroelectricity, where other ferroelectrics lose it [24].

**Figure 3:**
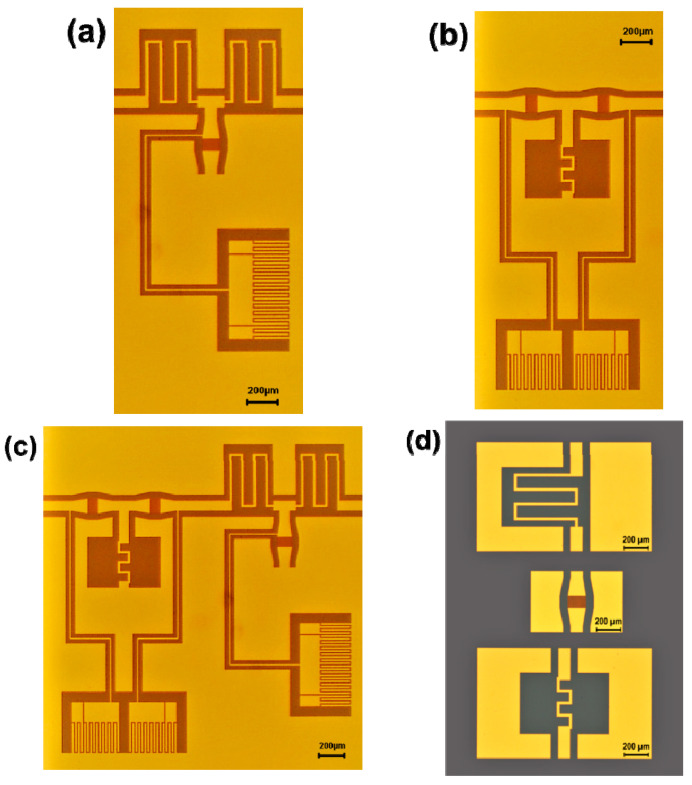
Optical pictures of the (**a**) low-pass filter, (**b**) high-pass filter, and (**c**) band-pass filter; and, (**d**) optical picture of the test structures. From top to down: inductor for low-pass filter, interdigital capacitor and inductor for high-pass filter.

The proposed ferroelectric filter needs fulfill some basic requirements: (i) low-voltage tunability; (ii) CMOS compatibility; (iii) compact dimensions; and, (iv) ease of fabrication, as mentioned in the Introduction. Moreover, a reconfigurability is envisaged to switch among the low-pass, the high-pass and the band-pass topologies. We describe the strategies adopted for each of the above constraints hereinafter. For the low-voltage tunability, as discussed in the Introduction we exploited the ferroelectric properties of HfZrO, which also confer CMOS compatibility and ease of integration, as a single mask is required to deposit the metal layer onto the HRSi/HfZrO substrate. The filters’ key sub-components are the inductors and the IDCs, with the latter working as variable capacitors (varactors) due to the underneath ferroelectric layer. For the reconfigurability, we chose to implement a BPF made of an LPF in series with an HPF. Between them, Single Pole Double Throw (SPDT) switches (to be integrated in the future) will allow the reconfiguration by selecting the desired filter (i.e., the appropriate input-output ports). The last requirement, perhaps the most critical one, is represented by the compact dimensions. For this purpose, the adopted strategy was to start with an equivalent circuit model (using AWR Microwave Office^®^) for each of the three filters and based on lumped elements. The next step was the translation of the three circuits into their 3D EM versions in CPW technology for ease of integration in order to take all the potential parasitic effects and EM couplings occurring in microwaves up to 20 GHz into account (using CST Microwave Studio^®^). Each basic filter (LPF and HPF) was implemented by a T-network; then, as already stated, the BPF was formed by connecting the LPF (made by two series inductances and a shunt varactor) to the HPF (made by two series varactors and a shunt inductor). As a major result, the BPF has much lower dimensions (3.19 mm × 3.47 mm) than the working wavelength. The optical images of the fabricated filters and the test structures are shown in Figure 3a–d. One can clearly distinguish the architectures of the three filters in Figure 3a–c, which are made of sub-components in CPW technology for ease-of-embedding in integrated circuits. One can also notice big interdigital adjustable capacitors for independent DC bias of the varactors, RF-decoupled by *λ_g_*/4-long lines (*λ_g_* is the guided wavelength at 10 GHz, i.e., the reference central frequency for the BPF). These capacitors have a capacitance of about 1 pF for the LPF and 0.3 pF for the HPF. Figure 3d displays the test structures of the filters’ key sub-components, i.e., the inductor for the LPF, the IDC, and the inductor for the HPF. In detail, the two series inductors of the LPF have a theoretical inductance of 0.79 nH, whereas the shunt inductor of the HPF has a theoretical inductance of 0.3 nH, in order to have the desired band-pass filtering in the X band (8.2–12.4 GHz). The gold has a thickness of 360 nm. Each IDC has 30 digits, which are 100 μm long and 2.5 μm wide, with a spacing of 2.5 μm between two adjacent digits. The overall IDC is only 147.5 μm wide.

## 3. Results and Discussion

The microwave measurements were made on-wafer at room temperature using a vector network analyser (VNA) Anritsu-37397D connected to a Karl-Süss PM5 probe station. The DC bias was provided by a DC source and the above mentioned big interdigital adjustable capacitors, in order to separate the DC bias from the high-frequency excitation signal.

### 3.1. Extraction of Relative Permittivity and Microwave Characteristics of HfZrO-Based IDCs

**Figure 4:**
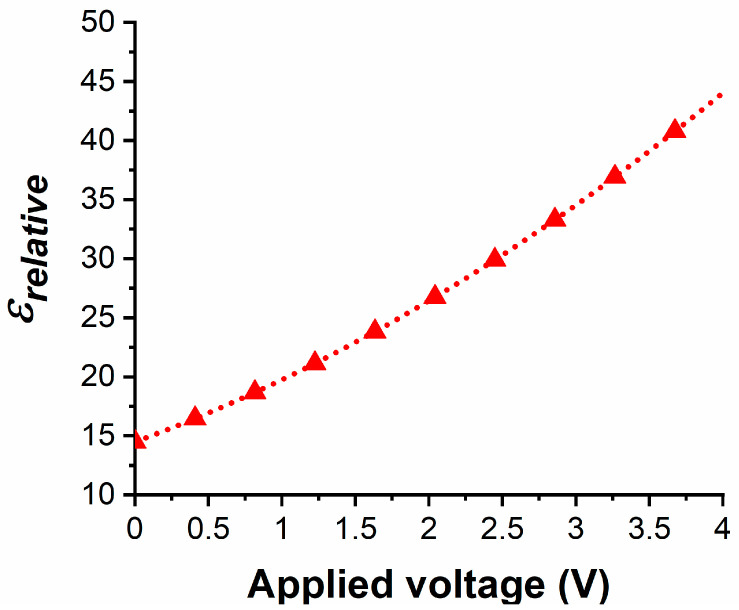
Extracted relative permittivity of the 6-nm-thick HfZrO ferroelectric at 10 GHz, as a function of the applied DC bias voltage in the range 0–4 V.

The extracted curve for *ε*(*V*) at 10 GHz using Equations (1) and (2) is shown in Figure 4, which was obtained by means of the Bianco–Parodi method described in [10] applied to two CPW lines of different lengths, designed on a HRSi/HfZrO substrate, and fabricated in the same run together with the structures that are presented in this work. Hence, this figure represents the value of the bias-dependent permittivity of HfZrO at a specific frequency, irrespective of the microwave device making use of a ferroelectric HfZrO thin film.

**Figure 5:**
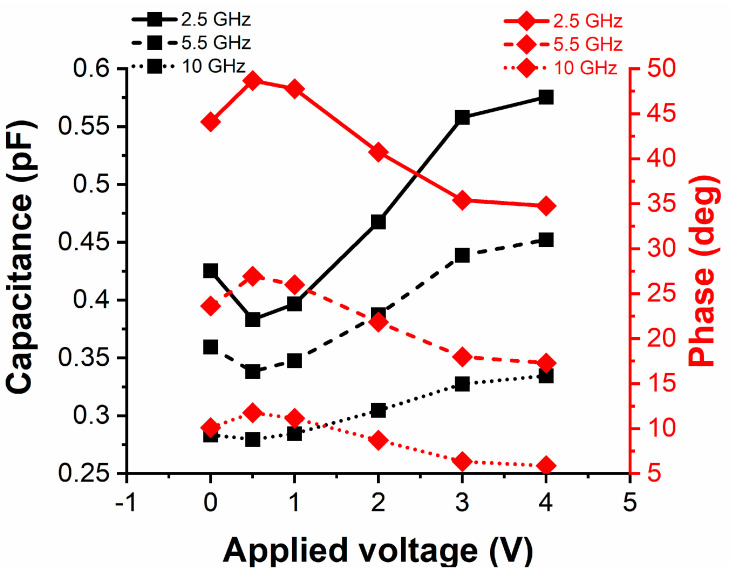
Measured capacitance (left vertical axis) and phase (right vertical axis) of the single interdigital capacitor at three reference frequencies (2.5 GHz, 5.5 GHz, and 10 GHz) for an applied DC bias voltage in the range 0–4 V.

Figure 5 displays the measurements for a single IDC in CPW configuration in terms of its capacitance *C_IDC_* and phase *ϕ_IDC_* (the latter as extracted from the transmission parameter |S_21_| or |S_12_|, which gives the proof in microwaves of the ferroelectricity of HfZrO) for three reference frequencies, i.e., 2.5 GHz and 5.5 GHz (associated to ISM—industrial, scientific, and medical—applications), and 10 GHz. We see that, at the frequency of 2.5 GHz, the sweeping of the DC voltage from 0.5 V up to 4 V increases the capacitance from 0.38 pF to 0.58 pF, whereas the phase decreases from 48.7° to 34.7°, i.e., a phase shift of 14°. Increasing the frequency to 10 GHz and sweeping the DC voltage in the same range, the capacitance increases from 0.28 pF to 0.33 pF, while the phase decreases from 11.76° to 5.86°, i.e., a phase shift of about 6°. This trend for *C_IDC_* and *ϕ_IDC_* is consistent with the ferroelectric nature of the HfZrO thin film: when applying an external DC electric field *E_a_*, the hysteric behaviour of the relative permittivity *ε* is such that it reaches its maximum *ε_max_* for *E_a,max_* ≠ 0 V/m [14] or, equivalently, for an applied DC bias voltage *V_max_* ≠ 0 V. In the case of HfZrO, *ε_max_* ≈ 60 at *V_max_* ≈ ±5 V. At the same time, the estimation of the capacitance of an IDC structure on a multi-layer substrate is quite cumbersome from a mathematical point of view, but, as expected, an increase of relative permittivity leads to an increase of the overall capacitance [25,26]. Also, the relation between *C_IDC_* and *ϕ_IDC_* is of the type *ϕ_IDC_* = *arctg*(1/*ωC_IDC_*) (where *ω* is the angular frequency); hence, an increase of the capacitance with the DC bias corresponds to a decrease of the phase. Table 1 summarises the main variations of *C_IDC_*, *R_IDC_* (extracted IDC’s resistance) and *ϕ_IDC_* at frequencies of interest. From Table 1 one can notice that *C_IDC_* can be increased for each frequency between 20% and 53% (with respect to its minimum value at 0.5 V), the results for the symmetric case—negative bias values—being very similar; this represents the key of the tunability characteristics of the proposed filter. At the same time, losses also increase with the DC bias (due to the increase of *R_IDC_*), which entails a higher insertion loss. However, as demonstrated in [10], the effective loss tangent of a 6-nm-thick HfZrO thin film decreases with frequency (and increases with bias). Nevertheless, it exhibits small variations with the applied DC voltage, which entails a lowering of the dielectric losses in the ferroelectric. Furthermore, a reduction of the skin depth is observed in metals when increasing the frequency, which also decreases the overall power losses of the EM waves that propagate along the single IDC. Possible countermeasures to reduce *R_IDC_* could be: (i) increase of the number of digits (using Electron Beam Lithography techniques); and, (ii) increase of gold thickness. These issues will be investigated in successive fabrication batches, as a trade-off between *C_IDC_* and *R_IDC_* is needed. Besides this, it is worth noting that a peculiar characteristic of any ferroelectric is its hysteretic nature. From the microwave point of view, when measuring the phase of the single IDC in the band of interest, the curve does not follow the same path when decreasing (or changing the polarity of) the applied DC voltage. In detail, when returning from ±4 V to 0 V, the reference (initial) state at 0 V was not re-established exactly or, in other words, a (temporary) change of the initial state has to be taken into account, together with a discharge process (with a recovery time in the order of a couple of minutes) that is necessary to re-align the ferroelectric dipoles in the unbiased situation.

### 3.2. Microwave Measurements of the Three HfZrO-Based Filters

**Figure 6:**
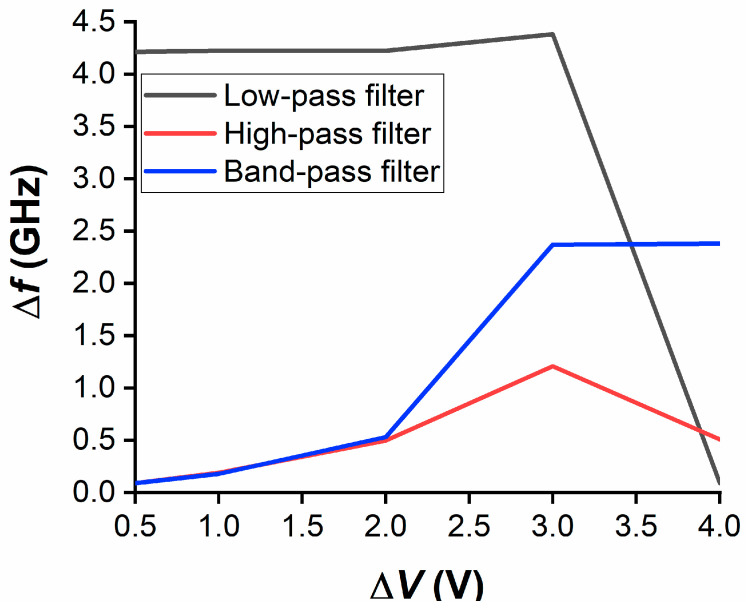
Frequency shift of the reflection coefficient |S_11_| for the three filters in the reference band, as a function of the applied DC bias voltage.

**Figure 7:**
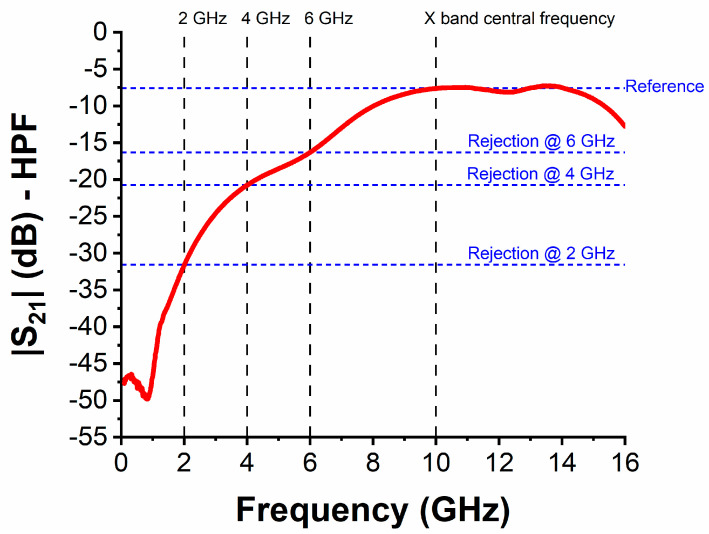
Transmission coefficient |S_21_| for the HPF at 3 V. The reference frequencies and values of |S_21_| are highlighted for an estimation at a glance of filter’s rejection properties.

Finally, the three filters were measured in the frequency range 0.1–16 GHz. Here, we stress that all three filters are deeply sub-wavelength (with overall dimension of about *λ*_0_/20 × *λ*_0_/10 for LPF and HPF, and of about *λ*_0_/9 × *λ*_0_/9 for BPF—where *λ*_0_ is the free-space wavelength at 10 GHz), which means that they exhibit higher losses than off-the-shelf bulk components or, in other words, miniaturized filters have the drawback of high losses which often require a post-amplification stage. Table 2 summarises the main performance for each one of the three filters in terms of |S_11_| and |S_21_|, whereas Figure 6 displays the frequency shift Δ*f* (in GHz) for |S_11_| of each filter in its reference band, as a function of the voltage difference Δ*V* = *V_i_* − *V_unbiased_* (in volts), where *i* = 1, 2, 3, 4, 5 and *V_unbiased_* = 0 V (unbiased case). The reference band is calculated when considering the frequency range in which |S_11_| is less than (or equal to) −6 dB, whereas the insertion loss (*IL*) is calculated as *IL* (dB) = −log_10_|S_21_|. The reference band for each filter was expected to cover partially (LPF) or entirely (HPF and BPF) the X band and, as one can notice from Table 2, this requirement is fully complied with. Table 2 also shows the minimum value of |S_11_| in the reference band and the best value of the *IL* in the X band (*IL_BEST,__X band_*). The *IL* exhibits high values due to the lossy nature of the three filters (as discussed before), especially for the BPF. Nevertheless, in the case of the BPF, the X band maximum rejection (defined as the difference between the maximum value of the *IL* and the *IL* at the edges of the X band, i.e., at 8.2 and 12.4 GHz) has a rather good value of more than 10.8 dB. Regarding Figure 6, it shows that the frequency shift for |S_11_| reaches almost 4.5 GHz at 3 V for the LPF, more than 1 GHz at 3 V for the HPF and more than 2.5 GHz at 4 V for the BPF. This frequency shift is the main effect that is given by the HfZrO ferroelectric thin film, since a change of maximum 8% of the effective permittivity (equivalent to a change of about 65% of the relative permittivity) allows for changing the phase of the microwave signal when passing through the filter (together with a moderate change of the characteristic impedance at filter’s ports, hence slightly improving or decreasing the matching to 50 Ω). When considering the performance of the three filters in terms of |S_21_| (|S_12_|), the most significant results were obtained for the HPF. In Figure 7, we show the transmission coefficient at 3 V (to which corresponds the maximum value of Δ*f* for the HPF in Figure 6, solid red curve): in this case, the rejection with respect to the central frequency in the X band (i.e., 10 GHz) is about 8.7 dB at 6 GHz, 13.2 dB at 4 GHz, and 24 dB at 2 GHz. Taking the previous discussion about the losses in the filter into account, it was expected to not have excellent values of steepness and rejection; these issues being presently under study in successive fabrication batches, including different dopants besides Zr.

## 4. Conclusions

In this paper, we have presented three ferroelectric-based microwave filters with low-voltage tunable characteristics, which allow for controlling the frequency shift of the reflection coefficient and the insertion loss in a wide frequency range, covering the band from 0.1 GHz up to 16 GHz. First, a simple yet effective mathematical relation has been derived in order to accurately predict the voltage-dependent behaviour of the relative permittivity of HfZrO ferroelectric thin films at a desired frequency, while taking its geometrical and physical characteristics into account. Subsequently, the fabricated 6-nm-thick HfZrO ferroelectric layer has been exploited for the design, fabrication, and experimental verification of a low-pass, a high-pass, and a band-pass filter embedding HfZrO-based varactors. In spite of the low value of the maximum DC bias applied (i.e., not exceeding 4 V), very promising tuning properties have been observed for all the filters under test, with a maximum frequency shift of almost 4.5 GHz at 3 V for the low-pass filter. These results further open the path towards low-voltage CMOS-compatible ferroelectric-based electronics, which are suitable for next-generation wireless applications.

## Figures and Tables

**Table 1:** Values of *C_IDC_* (pF), *R_IDC_* (Ω), and *ϕ_ID_*_C_ (deg) at different frequencies for two values of the DC bias voltage.

Frequency (GHz)	*C_IDC_* (pF)		*R_IDC_* (Ω)		*ϕ_IDC_* (deg)	
	0.5 V	4 V	0.5 V	4 V	0.5 V	4 V
**2.5**	0.38	0.58	76.43	85.53	48.69	34.76
**5.5**	0.34	0.45	65.69	73.13	26.94	17.29
**8**	0.30	0.38	59.51	65.70	17.27	10.14
**10**	0.28	0.33	55.2	60.46	11.76	5.86
**12**	0.26	0.31	51.67	56.19	7.05	2.05

**Table 2:** Main performance of the three filters in terms of reference band, minimum value of |S_11_| in the reference band, and best value of the insertion loss in the X band.

Filter Type	Reference Band	|S_11_|_min_	*IL_BEST,_* *_X band_*
**Low-Pass**	0.1–10.5 GHz	−22.8 dB	9.1 dB
**High-Pass**	4.13–16 GHz	−18.3 dB	7.5 dB
**Band-Pass**	4.6–15 GHz	−18.7 dB	14.7 dB
